# Identification and evolution of nsLTPs in the root nodule nitrogen fixation clade and molecular response of *Frankia* to AgLTP24

**DOI:** 10.1038/s41598-023-41117-1

**Published:** 2023-09-25

**Authors:** Mélanie Gasser, Jean Keller, Pascale Fournier, Petar Pujic, Philippe Normand, Hasna Boubakri

**Affiliations:** 1 Universite Claude Bernard Lyon 1, Laboratoire d′Ecologie Microbienne, UMR CNRS 5557, UMR INRAE 1418, VetAgro Sup, 69622 Villeurbanne, France; 2 LRSV, Université de Toulouse, CNRS, UPS, Toulouse INP, Castanet-Tolosan, France

**Keywords:** Phylogenetics, Plant evolution, Plant symbiosis, Bacterial transcription, Symbiosis, Peptides

## Abstract

Non-specific lipid transfer proteins (nsLTPs) are antimicrobial peptides, involved in several plant biological processes including root nodule nitrogen fixation (RNF). Nodulating plants belonging to the RNF clade establish symbiosis with the nitrogen-fixing bacteria rhizobia (legumes symbiosis model) and *Frankia* (actinorhizal symbiosis model) leading to root nodule formation. nsLTPs are involved in processes active in early step of symbiosis and functional nodule in both models. In legumes, nsLTPs have been shown to regulate symbiont entry, promote root cortex infection, membrane biosynthesis, and improve symbiosis efficiency. More recently, a nsLTP, AgLTP24 has been described in the context of actinorhizal symbiosis between *Alnus glutinosa* and *Frankia alni* ACN14a. AgLTP24 is secreted at an early step of symbiosis on the deformed root hairs and targets the symbiont in the nitrogen-fixing vesicles in functional nodules. nsLTPs are involved in RNF, but their functions and evolutionary history are still largely unknown. Numerous putative *nsLTPs* were found up-regulated in functional nodules compared to non-infected roots in different lineages within the RNF clade. Here, results highlight that nodulating plants that are co-evolving with their nitrogen-fixing symbionts appear to have independently specialized nsLTPs for this interaction, suggesting a possible convergence of function, which opens perspectives to investigate nsLTPs functions in RNF.

## Introduction

Root nodule nitrogen fixation symbioses (RNF) are established between plants belonging to the Fabales, Fagales, Cucurbitales, and Rosales orders and the nitrogen-fixing bacteria rhizobia and *Frankia*. In these mutualistic RNF, diazotrophic bacteria rhizobia establish symbioses with plants of the Fabales order and the genus *Parasponia* (Rosales). The filamentous actinobacteria *Frankia* have a wider spectrum, they establish symbiosis with Fagales, Cucurbitales, and Rosales comprising approximately 220 species^1^. These four plant orders form RNF clade grouping nodulating and non-nodulating plants. This distribution is likely due to the acquisition of nodulation by a common ancestor of the RNF clade, followed by multiple losses in the descendant lineages^2,3^. It should be noted that certain traits, such as haemoglobin, which is crucial for maintaining nitrogen fixation in the nodule, would have been gained after the acquisition of nodulation to adapt to the symbiont^3,4^. At the early steps of this interaction, the diazotrophic symbiont in contact with the plant roots enters in the plant tissue. Depending on the host plant, two modes of invasion are known: the intercellular infection and the intracellular infection via the root hairs, leading to nodule formation^5,6^. Into the nodule, the symbiont fixes atmospheric nitrogen and thus provides nitrogenous compounds to the plant, which in exchange transfers organic compounds derived from photosynthesis^7^. The recognition, entry, and maintenance of the bacterium in the nodule require fine coordination on the part of both partners, which is established through cellular pathways and molecular dialog between them. Studies of plant cellular mechanisms during nodulation have revealed the involvement of hosts’ secreted peptides classified as antimicrobial peptides (AMPs) to improve the interaction. In plants, AMPs are mainly described in the innate immune response of organisms to fight against biotic and abiotic stresses^8,9^. Their production by the host plant in a context of mutualistic symbiosis questions their biological roles in these interactions. In the two models of RNF symbiosis, three AMP families are described; the Nodule Cysteine Rich peptide (NCRs) and NCRs-like peptides secreted by Fabales plants of the IRLC and Dalbergioid clades^10,11^, the defensins secreted by actinorhizal plants^12–15^, and a third family investigated in this study, the non-specific lipid transfer protein (nsLTPs) secreted by nodulating plants belonging to the Fabales order and described in only one actinorhizal plant, *Alnus glutinosa*^16–19^. The nsLTPs are peptides with a hypervariable amino acid sequence of less than 100 residues and an N-terminal signal sequence that allows them to be addressed to target cell compartments as mature peptides^9^. They are characterized by 4 disulfide bridges formed by a conserved 8 Cysteines Motif (8CM) in the mature peptide: "C-X_n_-C-X_n_-CC-X_n_-CXC-X_n_-C-X_n_-C"^20^ where “X” represents any amino acid residue and “n” the number of amino acids. These disulfide bridges stabilize 4 alpha helices and give rise to a hydrophobic tunnel-like cavity allowing the binding and transport of hydrophobic molecules^21^. This structure allows them to resist heat, denaturing agents, and proteases^22^. This AMPs family is widely distributed in plant tissues among all land plants suggesting that nsLTPs were originally acquired in their common ancestor^23,24^. They may have been gained even earlier, as a putative nsLTPs in a green alga was predicted^25^. In plants, nsLTPs are involved in plant innate immunity and are classified as pathogenesis-related proteins (PR-14) but are also involved in several biological processes such as stress resistance, reproduction, germination, plant defense against pathogen attacks, cuticle formation, pollen tube formation, and RNF symbiosis^17–19,21,26^.

In RNF symbiosis, nsLTPs were first described in legumes (Fabales) at the early step of nodulation and in nodules of *Medicago truncatula*, *Astragalus sinicus* (Chinese milk vetch), and *Phaseolus vulgaris*^24,27,28^. The nsLTPs MtN5 and MtLTP7 are secreted by *M. truncatula* to regulate symbiont entry into the root epidermis and promote infection in the root cortex^16,17,27–29^. In *A. sinicus* a nsLTPs named AsE246 is also expressed at early and late steps of nodulation and is localized on the symbiosome membrane, which could be involved in membrane biosynthesis and to promote symbiosis efficiency^19^. Little is known about the involvement of AMPs during actinorhizal symbiosis due to the lack of genetic tools. However, a transcriptomic analysis at early and maturing steps of nodulation permitted to identify a gene encoding an nsLTPs up-regulated in deformed root hairs and functional nodule of *A. glutinosa* in symbiosis with *Frankia alni* ACN14a compared to non-infected roots^18^. This peptide named AgLTP24 is addressed to deformed root hairs at an early step of symbiosis and targets the nitrogen-fixing vesicle cells of *Frankia* at a later step in nodules. As AgLTP24 targets the symbiont in the nodule, the effect of this peptide on the physiology of *Frankia* was tested in a previous article and showed that high concentration (5 µM) decreased metabolic activity and lower concentration (100 nM) inhibited nitrogen fixation^18^.

This study aimed to retrace the evolutionary history of nsLTPs in RNF symbiosis as they are involved in both symbiotic models. For this purpose, putative nsLTPs were identified in proteomes of nodulating and non-nodulating plants belonging to the RNF clade and the differential expressions of *nsLTPs* in nodules for five nodulating plants of the four orders were retrieved from available transcriptomics data. We showed that the nsLTPs family was widespread in nodulating plants as *nsLTPs* genes were expressed in the functional nodules (which are nodules with an active nitrogen fixation activity) of plants belonging to the four orders. Regarding their evolution in relation to the RNF symbiosis, this family would have been independently co-opted in different lineages suggesting a possible convergence of function. It is important to note that nsLTPs must share the same function in RNF symbiosis to show convergence, so more functional studies are required to conclude on this point. To deepen our understanding of their function during symbiosis, AgLTP24 secreted by *A. glutinosa* was further studied by investigating the molecular response of the symbiont *F. alni* ACN14a to subinhibitory concentrations of this nsLTP.

## Results

### nsLTPs identification and characterization

**Figure 1:**
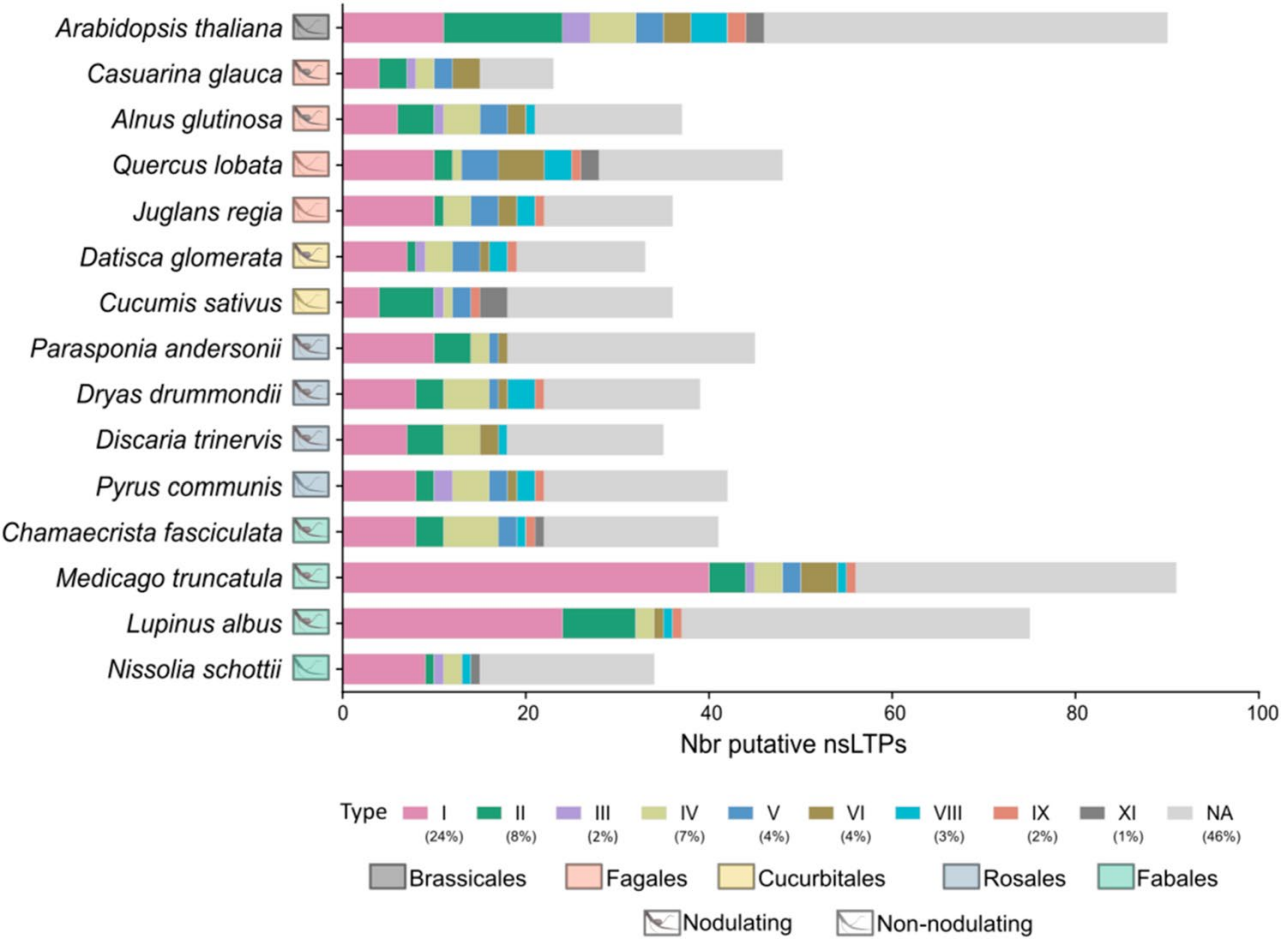
Putative nsLTPs characteristics. Graphical representation of the number of putative nsLTPs retrieved in plants proteomes belonging to the RNF clade and *A. thaliana*. Plant orders are represented with colored boxes on the y-axis. Nodulating and non-nodulating plants are identified by a schematic representation of a root with or without a nodule. The number of nsLTPs for each plant is represented by the number of typed and untyped (NA) nsLTPs. The percentages of the different types of nsLTPs in this dataset are shown in the legend.

The identification of nsLTPs was done using 15 proteomes of nodulating and non-nodulating plants distributed in the RNF clade and *Arabidopsis thaliana* belonging to the Brassicales order (Fig. 1).

The nsLTPs are characterized by a hypervariable amino acid sequence and an N-terminal signal sequence. The signal sequence that is responsible for facilitating peptide secretion, which is cleaved during the secretion, resulting in the generation of mature peptides. The mature nsLTPs possess a conserved 8-cysteine motif (8CM) “C-X_n_-C-X_n_-CC-X_n_-CXC-X_n_-C-X_n_-C”, where 'X' represents any amino acid residue and 'n' the number of amino acids. Due to the sequence hypervariability, conventional approaches such as keyword searches and BLAST analyses are not suited for exhaustively retrieving these peptides^9^. To identify nsLTPs a wrapper script, nsLTPFinder, was made to identify proteins containing an N-terminal signal peptide, with a mature sequence containing a conserved 8 Cysteine Motif (8CM), characteristic of this peptide family.

From the 15 proteomes, an overall number of 705 putative nsLTPs was identified ranging from 23 in the *Casuarina glauca* proteome to 91 in the *Medicago truncatula* proteome (Fig. 1 and Supplementary Table S1). Plants belonging to the Fagales order had between 23 and 48 putative nsLTPs, those belonging to the Cucurbitales between 33 and 36, the Rosales had between 35 and 45 putative nsLTPs and the Fabales between 34 and 91 (Fig. 1). The number of putative nsLTPs in plant proteomes was compared with non-parametric Mann–Whitney tests as the data do not follow a normal distribution (Shapiro test) and all *p*-values were above the threshold of 0.05. This indicated that the number of putative nsLTPs in plant proteomes was not significantly different across the 5 different plant orders nor different based on the capability of the plant to establish RNF symbiosis. Plant proteomes used in this study did not have the same annotation level, thus, the number of nsLTPs predicted for these proteomes are subject to change with the increasing number of genome sequencing or proteomic studies.

Putative nsLTPs were then grouped according to the classification proposed by Boutrot et al.^20^ with the addition of the XI type proposed by Li et al.^30^ (Fig. 1, Supplementary Table S1). Only 54% of the putative nsLTPs in this dataset could be classified; the most represented was type I with 24% and type VII was not retrieved in our data. The absence of the type VII in our data can be attributed to its specificity to monocotyledons^31^. Among the 326 nsLTPs unassigned to a type, some had a large domain rich in proline, aspartic acid and histidine composed of more than 40 amino acids between the second and third cysteine of the 8CM. This lack of assignation underlines the fact that the current classification is not exhaustive.

### nsLTPs expression during nodulation and evolution history in nodulating plants

**Figure 2:**
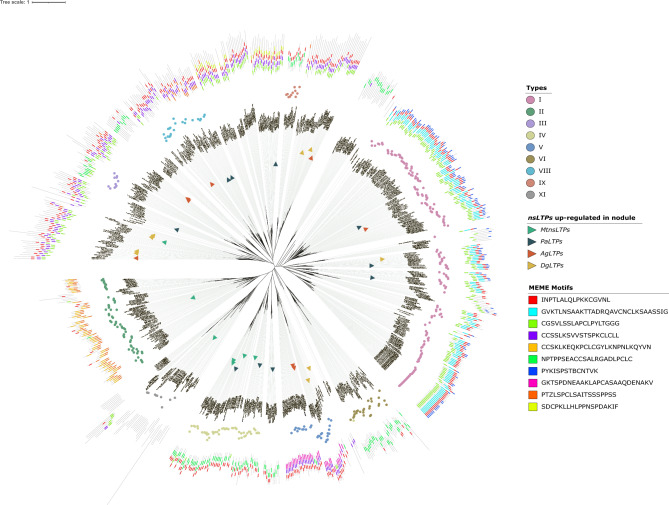
Phylogenetic tree representing nsLTPs evolution in the RNF clade. A graphical representation of the unrooted maximum-likelihood phylogenetic tree of nsLTPs from RNF plants was constructed with IQ-TREE. Typed nsLTPs are represented by colored circles. The unannotated ones are those that do not correspond to any type. The triangles represent the *nsLTPs* that are up-regulated in functional nodules, of *M. truncatula (MtnsLTPs), P. andersonii (PaLTPs), A. glutinosa (AgLTPs) and D. glauca (DgLTPs).* For *A. glutinosa*, *AgLTPs* up-regulated in nodules compared to non-infected roots are represented by qRT-PCR data (this study). For the tree other plant, up-regulated *MtnsLTPs*, *PaLTPs* and *DgLTPs* in functional nodules were retrieved from databases (see “Materials and methods”). Names of nsLTPs already described in the literature as involved in RNF symbiosis are specified next to the gene name. The scale bar represents the number of substitutions per site (under the selected evolutionary model). The schematic representation of conserved protein motifs predicted using the MEME suite is shown outside the tree.

The phylogeny of nsLTPs was assessed using putative nsLTPs identified in nodulating and non-nodulating plants belonging to the four orders of the RNF clade and *A. thaliana* (Brassicales order), as an outgroup. It is worth noting that the clades are more representative of the different types of nsLTP than of the different plant orders. Furthermore, within each plant order, different types of nsLTP were found. In the phylogenetic tree, nsLTPs of all plants were present in all clades suggesting that they would have undergone several duplication events. The untyped nsLTPs were grouped in phylogenetic clades with typed nsLTPs and shared the same conserved protein motifs (Fig. 2).

A conserved protein motif analysis using the MEME suite was conducted and showed that the majority of nsLTPs grouped in the same clade and mostly shared the same conserved protein motifs (Fig. 2).

We then identified nsLTPs that might be involved in symbiosis and investigated their distribution in the phylogenetic tree. For that, the expression levels of *nsLTPs* in functional nodules were retrieved from published transcriptomic data for five plants belonging to the four orders of the RNF clade: *Medicago truncatula* (Fabales), *Parasponia andersonni* (Rosales), *Datisca glomerata* (Cucurbitales), *Alnus glutinosa* and *Casuarina glauca* (Fagales). For *A. glutinosa* in association with *Frankia alni* ACN14a, transcriptomic data based on EST microarrays were complemented in this study using qRT-PCR targeting 23 genes encoding putative nsLTPs (AgLTPs) to characterize differential expression in the nodule compared to non-infected roots.

**Figure 3:**
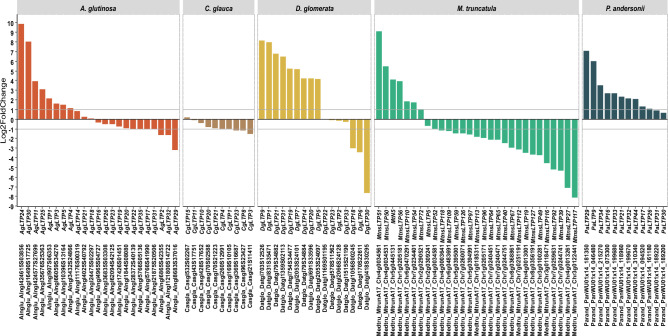
Differential expression of genes encoding putative nsLTPs in functional nodules. Graphic representation of differential expressions of the genes encoding nsLTPs at nodule stage compared to control conditions. The x-axis carries the gene name and the nsLTP annotation, on the y-axis expression level is in Log2FoldChange (up-regulated: Log2FC ≥ 1 and down-regulated: Log2FC ≤ -1). For *A. glutinosa*, differential expression of *AgLTPs* in nodules is represented by qRT-PCR data (this study). Differential expressions of *nsLTPs* were retrieved from databases (see “Materials and methods”).

Up- and down-regulated genes encoding nsLTPs in functional nodules were identified in plants belonging to the 4 orders of the RNF clade (Log2FoldChange ≥ 1 or ≤ − 1) (Fig. 3).

The databases used (see “Materials and methods”), enabled the identification of the differential expression of 26% to 62% of genes encoding nsLTPs within the functional nodules compared to non-infected roots. In nodules of the Fabales *M. truncatula* and the Rosales *P. andersonni* capable of establishing symbioses with rhizobia, 7 and 10 up-regulated and 1 and 2 not-regulated *nsLTPs* were retrieved, respectively. They were no down-regulated *nsLTPs* found for *P. andersonni* while 21 *nsLTPs* were down-regulated in *M. truncatula* nodules. Concerning actinorhizal plants, *D. glomerata* had at the nodule step, 9 up-regulated, 4 not-regulated and 3 down-regulated *DgLTPs*. For the Fagales, *C. glauca*, no *CgLTPs* was up-regulated, 5 were not-regulated and 5 *CgLTPs* were down-regulated in nodule. For *A. glutinosa*, previous transcriptomic data based on EST microarrays led to the identification of four genes (corresponding to 4 ESTs) up or down regulated in the nodule compared to non-infected roots (Log2FoldChange ≥ 1 or ≤ − 1) (see Supplementary Table S2). One of these EST matches with two putative *AgLTPs* (*AgLTP1* (Alngl907S06353) and *AgLTP3* (Alngl66059S34270)) with a high percentage of identity. Thus, for *A. glutinosa* in association with *F. alni* ACN14a EST data were refined using qRT-PCR targeting genes encoding putative AgLTPs. The expressions of 23 *AgLTPs,* including 8 up-regulated, 8 not-regulated and 7 down-regulated (Log2FoldChange ≥ 1 or ≤ − 1), in the functional nodule compared to uninfected roots were assessed and the differential expression of *AgLTP1* and *AgLTP3* was refined (Fig. 3 and Supplementary Table S2). We also confirmed that *AgLTP24* (Alngl424615S03856) is the most up-regulated gene encoding an nsLTP at the functional nodule step.

The nsLTPs described as involved in symbiosis in the literature and those up-regulated in the functional nodule were retrieved in different phylogenetic clades, had different conserved protein motifs and different isoelectric points and molecular weights (Fig. 2 and Supplementary Table S1). MtN5 (MtrunA17_Chr5g0445131), AgLTP24 and MtnsLTP54 (MtrunA17_Chr7g0234401), (The annotation of MtnsLTPs was done according to the one proposed in the LEGOO database : MtnsLTP54 corresponds to MtLTP7 described by Santi et al.^28,32,33^) already described as involved in RNF symbiosis were grouped in the same phylogenetic clade with the same conserved protein motif predicted by MEME. MtN5 and AgLTP24 mature peptides had close isoelectric points (IP) and molecular weights but shared only 21% of sequence identity^18^. MtN5 and MtnsLTP54 mature peptides had a higher sequence identity (38%) but a different molecular property with an IP of 8.8 and 4.4, respectively (see Supplementary Table S1). Overall, these results indicate that *nsLTPs* up-regulated in functional nodules compared to uninfected roots have diverse protein motifs in their protein sequence and that nsLTPs described in the literature as functionally involved in symbiosis share the same conserved protein motifs.

### Molecular response of *Frankia alni* ACN14a to AgLTP24

To further investigate the role of nsLTPs in symbiosis, we investigated the function of AgLTP24 secreted by *A. glutinosa* in symbiosis with *F. alni* ACN14a. Our previous work demonstrated that *AgLTP24* was highly expressed in *A. glutinosa* both at an early step of infection with *F. alni* ACN14a and at the functional nodule step^18^. In planta, AgLTP24 is secreted at deformed root hairs during the early step of symbiosis when the host recognizes *Frankia* and later when it targets the nitrogen-fixing vesicles of the symbiont inside the nodule cells. This previous work has showed that AgLTP24 at 5 µM impacted *Frankia* physiology by inhibiting cellular activity and nitrogen fixation at 100 nM and above. As *Frankia* in the nodule is viable and has an active nitrogen fixation to provide nitrogen to the plant, we investigated the molecular response of *F. alni* ACN14a under N-free conditions in contact or not with a sub-inhibitory concentration of AgLTP24 (1 nM) using RNAseq method (Table 1).

**Table 1:** *Frankia alni* ACN14a genes up and down regulated in N-free condition supplemented with AgLTP24 versus N-free condition.

Label	Name	Log2 FoldChange	p_adj_	Annotation
FRAAL1882		5.01	5.57E−03	Small heat shock protein
FRAAL4716		4.14	3.89E−02	Putative oxidoreductase
FRAAL6701	groL	4.10	4.24E−04	Chaperone Hsp60 (GroEL), part of GroE chaperone system
FRAAL4715		3.87	3.95E−02	Putative transcriptional regulator of the TetR family
FRAAL1884	ribD	3.55	1.10E−03	Putative riboflavin/cytosine deaminase (partial)
FRAAL0166		3.46	4.42E−03	Conserved hypothetical protein; putative membrane protein
FRAAL5655	sdhB	3.40	4.90E−03	Succinate dehydrogenase iron-sulfur protein
FRAAL1764	lon	3.29	2.11E−03	DNA-binding ATP-dependent protease La; heat shock K-protein
FRAAL2325		3.23	4.90E−03	putative 3-(3-hydroxy-phenyl)propionate hydroxylase, FAD/NAD(P)-binding
FRAAL5654	sdhA	3.10	5.06E−03	Succinate dehydrogenase flavoprotein subunit A
FRAAL6439		3.07	1.30E−02	Putative MoxR-like regulatory protein
FRAAL5653	sdhC	3.04	5.57E−03	Succinate dehydrogenase cytochrome B subunit
FRAAL6438		2.95	8.43E−03	Hypothetical protein
FRAAL6814	nifV	2.94	4.90E−03	Nitrogenase-associated homocitrate synthase
FRAAL5037	livG	2.88	7.01E−03	High-affinity branched-chain amino acid transport protein (ABC superfamily, atp_bind)
FRAAL6643	clpB	2.82	4.90E−03	ATP-dependent protease, Hsp 100, part of multi-chaperone system with DnaK, DnaJ, and GrpE
FRAAL0168		2.81	1.56E−02	Hypothetical protein; putative signal peptide; putative Dyp-type peroxidase domain
FRAAL6804	nifZ	2.79	5.06E−03	NifZ protein
FRAAL4308	copD	2.79	7.90E−03	Copper resistance membrane protein
FRAAL2326		2.76	2.55E−02	Hypothetical protein; putative serine-threonine protein kinase
FRAAL0988		2.69	5.06E−03	Putative regulator
FRAAL6807		2.62	4.62E−03	Conserved hypothetical protein
FRAAL6813	nifH	2.62	1.11E−02	Nitrogenase iron protein (NITROGENASE component II) (nitrogenase Fe protein) (nitrogenase reductase, dinitrogenase reductase)
FRAAL1134	groL	2.61	5.55E−03	Chaperone Hsp60 (GroEL), part of GroE chaperone system
FRAAL0989		2.61	1.16E−02	Cation-transporting P-type ATPase A
FRAAL6812	nifD	2.60	9.91E−03	Nitrogenase molybdenum-iron protein alpha chain (nitrogenase component I) (Dinitrogenase)
FRAAL6811	nifK	2.59	1.65E−02	Nitrogenase molybdenum-iron protein beta chain (nitrogenase component I) (dinitrogenase)
FRAAL1133	groS	2.58	6.17E−03	Chaperone Hsp10 (GroES), part of GroE chaperone system
FRAAL6638		2.57	1.41E−02	Hypothetical protein
FRAAL6640	grpE	2.56	1.52E−02	Heat shock protein (HSP-70 cofactor)
FRAAL4648		2.50	3.88E−02	(2,3-dihydroxybenzoyl)adenylate synthase (2,3-dihydroxybenzoate-AMP ligase; Dihydroxybenzoic acid-activating enzyme)
FRAAL4645	cetJ2	2.48	2.55E−02	Cupin domain-containing protein; fralnimycin synthesis
FRAAL6859		2.47	1.43E−02	Cupin domain-containing protein; fralnimycin synthesis
FRAAL4644	cetJ3	2.42	2.98E−02	Conserved hypothetical protein
FRAAL2286		2.42	1.16E−02	Putative WhiB-family transcriptional regulator; putative role in cell cycle control
FRAAL6639	dnaK	2.41	2.01E−02	Chaperone Hsp70 in DNA biosynthesis/cell division
FRAAL5036		2.38	1.21E−02	Putative high-affinity branched-chain amino acid transport protein (ABC superfamily, atp_bind)
FRAAL1506		2.36	4.90E−03	Hypothetical protein
FRAAL4245		2.35	7.01E−03	Hypothetical integral membrane protein
FRAAL1607		2.34	4.35E−02	Putative integral membrane protein
FRAAL6437		2.34	1.64E−02	Putative transglutaminase, putative cysteine proteases, putative membrane protein
FRAAL5911		2.33	1.63E−02	Hypothetical protein; putative signal peptide
FRAAL6335		2.32	2.91E−02	Hypothetical protein
FRAAL6641	dnaJ	2.32	1.98E−02	Heat shock protein (Hsp40), co-chaperone with DnaK
FRAAL6197		2.28	4.92E−02	Hypothetical protein; putative signal peptide
FRAAL6808	nifX	2.26	4.59E−02	NifX protein
FRAAL0596		2.20	4.24E−04	Putative regulator
FRAAL1002		2.13	3.31E−02	Putative cytochrome C biogenesis membrane protein
FRAAL6802	erpA	2.13	2.19E−02	Conserved hypothetical protein; Thioredoxin-like domain
FRAAL6860		2.13	3.70E−02	Putative 1L-myo-inositol-1-phosphate synthase
FRAAL0060		2.11	1.21E−02	Putative transcription regulator protein
FRAAL6803	nifB	2.08	2.09E−02	FeMo cofactor biosynthesis protein nifB
FRAAL1763		2.07	2.19E−02	Hypothetical protein
FRAAL6337		2.06	1.68E−02	Hypothetical protein
FRAAL5912	sigE	2.01	4.97E−02	Putative RNA polymerase ECF-subfamily sigma factor
FRAAL1001		1.98	2.55E−02	Thiol:disulfide interchange protein helX precursor (Cytochrome c biogenesis protein helX)
FRAAL4136		1.98	3.31E−02	Putative iron sulphur protein (Putative secreted protein)
FRAAL4747		1.97	2.80E−02	Putative stress-inducible protein; putative adenine nucleotide-binding domain
FRAAL6642		1.97	1.72E−02	Putative heat shock protein hspR
FRAAL6118		1.91	1.43E−02	Putative glycosyltransferase
FRAAL5016		1.91	4.59E−02	Hypothetical protein
FRAAL5461		1.82	2.25E−02	Putative Epoxide hydratase
FRAAL5462		1.80	5.06E−03	Putative TetR-family transcriptional regulator
FRAAL3287		1.76	2.20E−02	Glycine-rich cell wall structural protein
FRAAL1786		1.74	2.20E−02	Hypothetical protein
FRAAL4148		1.73	2.91E−02	Hypothetical protein
FRAAL3704		1.73	4.65E−02	Putative TetR-family transcriptional regulator
FRAAL5121		1.70	4.15E−02	Putative integral membrane protein
FRAAL4491	oxyR	1.69	5.06E−03	Transcriptional regulator of oxidative stress, regulates intracellular hydrogen peroxide (LysR family)
FRAAL4781		1.68	4.22E−02	Conserved hypothetical protein
FRAAL0075		1.67	2.98E−02	Conserved hypothetical protein
FRAAL3899		1.66	1.98E−02	Putative ATP/GTP binding protein; putative beta WD-40 repeat and TPR domains
FRAAL0863		1.66	1.21E−02	RicinB lectin
FRAAL0081		1.65	1.21E−02	Manganese transport system ATP-binding protein
FRAAL1576		1.63	4.22E−02	Hypothetical protein; putative signal peptide
FRAAL6408		1.63	5.06E−03	Conserved protein of unknown function
FRAAL6119		1.62	1.64E−02	Putative succinoglycan biosynthesis protein
FRAAL5109	qcrB	1.61	2.19E−02	Ubiquinol-cytochrome c reductase cytochrome b subunit
FRAAL1508		1.59	5.06E−03	Hypothetical protein; putative membrane protein
FRAAL5649		1.59	1.21E−02	Integral membrane protein with Succinyl-CoA ligase domain
FRAAL6227		1.52	1.86E−02	Putative NADH dehydrogenase
FRAAL5552		1.51	2.58E−02	Hypothetical protein; putative signal peptide
FRAAL4430		1.47	1.21E−02	Hypothetical protein; putative signal peptide
FRAAL1766		1.45	3.88E−02	Putative pirin-like protein
FRAAL1505	murI	1.43	2.98E−02	Glutamate racemase
FRAAL0938		1.43	3.56E−02	Putative molybdopterin converting factor
FRAAL1699	groL	1.39	1.56E−02	Chaperone Hsp60 (GroEL), part of GroE chaperone system
FRAAL4024		1.38	2.79E−02	Putative monooxygenase with luciferase-like ATPase activity
FRAAL1427		1.35	4.97E−02	Short-chain dehydrogenase/oxidoreductase with several Glucose/ribitol dehydrogenase and 17-Beta hydroxysteroid dehydrogenase domains
FRAAL6502		1.35	6.17E−03	conserved hypothetical protein
FRAAL1154	accD	1.33	2.55E−02	Acetyl-coenzyme A carboxylase carboxyl transferase subunit beta (ACCASE beta chain)
FRAAL0924		1.33	4.97E−02	Putative MarR-family transcriptional regulator; putative signal peptide
FRAAL2626		1.33	1.43E−02	Hypothetical protein
FRAAL5351		1.31	4.90E−02	Putative Na + /H + antiporter; putative membrane protein
FRAAL5685		1.29	4.45E−02	Membrane-bound Ribonuclease BN
FRAAL5137		1.29	1.88E−02	Hypothetical protein
FRAAL4890		1.25	2.19E−02	Putative transcriptional regulator (partial match)
FRAAL1577		1.24	3.56E−02	Short-chain dehydrogenase, NAD(P)-binding domain
FRAAL1016		1.23	1.65E−02	Hypothetical protein
FRAAL0229		1.22	4.57E−02	Hypothetical protein
FRAAL6260		1.22	2.55E−02	Secreted subtilisin-like serine protease
FRAAL2542		1.21	2.01E−02	Non-ribosomal peptide synthetase
FRAAL1157	sucD	1.13	3.88E−02	Succinyl-CoA synthetase, alpha subunit, NAD(P)-binding
FRAAL5156	lipB	1.11	4.85E−02	Lipoyltransferase (Lipoyl-[acyl-carrier protein]-protein -N-lipoyltransferase) (Lipoate-protein ligase B)
FRAAL5646	fecD	1.02	4.97E−02	Citrate-dependent iron (III) transport protein (ABC superfamily, membrane)
FRAAL4818		1.02	1.16E−02	Hypothetical protein
FRAAL6723		1.01	3.51E−02	Hypothetical protein; putative ATPase domain
FRAAL3352		− 1.02	1.63E−02	Putative phosphatidylinositol diacylglycerol-lyase
FRAAL0541		− 1.05	2.19E−02	Putative dehydrogenase/oxidoreductase
FRAAL0375		− 1.11	2.97E−02	Putative GntR-family transcriptional regulator
FRAAL0640		− 1.13	1.21E−02	Putative secreted cell wall peptidase
FRAAL2594		− 1.16	1.98E−02	Putative epoxide hydrolase
FRAAL1590		− 1.19	2.80E−03	Putative conserved protein; glyoxalase and dihydroxybiphenyl dioxygenase domain
FRAAL2705		− 1.19	2.98E−02	Hypothetical protein
FRAAL6479		− 1.20	3.47E−02	Short− chain dehydrogenase
FRAAL3995		− 1.22	3.58E−02	Putative hydrolase
FRAAL2484		− 1.32	2.19E−02	Conserved hypothetical protein
FRAAL3997		− 1.38	7.64E−03	Conserved hypothetical protein
FRAAL4133		− 1.42	3.45E−02	Putative glutathione S-transferase enzyme with thioredoxin-like domain
FRAAL2519		− 1.43	3.09E−02	Putative short-chain dehydrogenase
FRAAL2937		− 1.45	2.25E−02	Hypothetical protein
FRAAL3838		− 1.46	4.97E−02	Hypothetical protein; putative signal peptide
FRAAL3585		− 1.48	3.31E−02	Putative esterase
FRAAL3774		− 1.54	2.55E−02	hypothetical protein
FRAAL1571		− 1.57	1.64E−02	Putative acyl-CoA dehydrogenase
FRAAL0744		− 1.59	5.55E−03	Hypothetical protein; putative endonuclease domain
FRAAL2938		− 1.62	8.43E−03	Putative protein kinase
FRAAL3965		− 1.63	2.11E−02	Hypothetical protein; putative signal peptide
FRAAL2509		− 1.63	3.31E−02	Putative 3-ketoacyl-CoA thiolase
FRAAL0201		− 1.67	2.98E−02	Putative cytochrome P450 reductase
FRAAL3923		− 1.69	4.22E−02	Putative cytochrome P450
FRAAL0376		− 1.71	1.88E−02	Cytosine/purine/uracil/thiamine/allantoin permease family protein
FRAAL4527		− 1.71	5.57E−03	Putative Glycoside hydrolase
FRAAL2508		− 1.73	2.63E−02	Protein associated with acetyl-CoA C-acyltransferase
FRAAL0316		− 1.76	1.43E−02	Carveol dehydrogenase
FRAAL0200		− 1.87	6.87E−04	NAD+-dependent aldehyde dehydrogenase
FRAAL2706		− 1.89	1.98E−02	Hypothetical protein
FRAAL0348		− 1.90	1.30E−02	Putative 6-methylsalicylic acid synthase
FRAAL2076	narK	− 2.00	2.91E−02	Nitrite membrane extrusion protein
FRAAL2707		− 2.00	1.43E−02	Coenzyme PQQ synthesis protein
FRAAL2513		− 2.00	2.19E−02	Putative Acyl-CoA dehydrogenase
FRAAL3766		− 2.65	1.52E−02	Hypothetical protein

*p*-value adjusted (p_adj_) ≤ 0.05, up-regulated genes: Log2FoldChange ≥ 1, Down-regulated genes Log2FoldChange ≤  − 1. Gene label, name and annotation come from the Genoscope database.

Physiological measurements such as nitrogen fixation (ARA), respiration (IRA), and growth (OD_600nm_) were conducted on these assays and confirmed that AgLTP24 at this concentration did not affect *Frankia* physiology as shown earlier (see Supplementary Fig. S1)^18^.

Transcriptomic analysis identified 107 up-regulated genes and 35 down-regulated genes (Table 1) when *F. alni* ACN14a was in contact with a sub-inhibitory concentration of AgLTP24 compared to the control condition. Some encoded chaperones involved in refolding proteins and proteins for repairing DNA damages were up-regulated, such as *groL*, *groS, lon,* and a gene cluster (FRAAL6639-FRAAL6643) with *dnaK*, *grpE*, *dnaJ*, and *clpB*. Genes encoding putative proteins involved in cell wall/membrane/envelope biogenesis were up-regulated such as FRAAL6118 and FRAAL6119 encoding a glycosyltransferase and a succinoglycan biosynthesis protein, respectively. Other upregulated genes encoding membrane transporters such as ABC transporters, manganese transport, cation transporting P-type ATPase A, and a citrate transporter (*fecD)* were retrieved. Several genes involved in energy conversion and metabolism, aerobic respiration (succinate dehydrogenase sdhC, sdhA, sdhB), cytochromes, and nitrogen fixation (*nif* genes*, nifB, nifK, nifX, nifH, nifV, nifZ, nifD*) were up-regulated while *narK* allowing nitrate and nitrite import was repressed. Among the down-regulated genes, only one gene was annotated (*narK*), the others were not described enough to provide further information.

## Discussion

Plant AMPs are involved in many plant functions, such as innate immunity or RNF symbiosis. Some legumes belonging to the IRLC and Dalbergioids clades secrete NCRs and NCR-like respectively to coordinate the terminal differentiation of rhizobia into polyploid bacteroids in the nodule. These NCRs and NCR-like are characterized by a conserved cysteine motif in their protein sequence that is close to the cysteine motif of defensins and neurotoxins. In actinorhizal symbioses, less information is available due to the lack of genetic engineering tools developed. Based on transcriptomic analysis of nodules, AMPs of the defensins family have been identified in the three actinorhizal plants *Ceanothus thrysiflorus* (Rosales), *D. glomerata* (Cucurbitales), and *A. glutinosa* (Fabales). These in silico analyses were complemented with functional analyses of AgDef5, a defensin secreted by *A. glutinosa* at the early step of symbiosis and in the nodule. In vitro, AgDef5 permeabilizes *F. alni* ACN14a’s nitrogen-fixing vesicles, leading to the leakage of nitrogen-rich metabolites, which could improve trophic exchanges between the two partners in planta^34^.

The nsLTPs family is involved in RNF symbiosis in both rhizobia/legumes and *Frankia/*actinorhizal symbioses*.* These peptides are secreted early in the symbiosis and in the nodule. In *P. vulgaris*, nsLTPs have a putative role and possible interaction with respiratory burst of oxidase homologs (RBOH)-dependent reactive oxygen species (ROS) production^24^. In *M. truncatula* and *A. sinicus*, MtN5 and AsE246 respectively, could regulate symbiont invasion, promote root cortex entry, membrane biosynthesis, and symbiosis efficiency^16,17,19,27–29^. In actinorhizal symbiosis, only one nsLTP has been studied in *A. glutinosa*, AgLTP24, which targets *Frankia*’s nitrogen-fixing vesicle in nodules^18^. Purified AgLTP24 peptide inhibited *F. alni* ACN14a nitrogen fixation activity above 100 nM and reduced metabolic activity above 5 µM in vitro.

As this family is widespread in RNF symbiosis, the evolutionary history of nsLTPs in symbiosis was analyzed in this study. First, nsLTPs were predicted using 15 plant proteomes of nodulating and non-nodulating plants within the RNF clade and one Brassicales *A. thaliana*. These data permit to perform a phylogenetic analysis, which showed that *nsLTPs* from diverse plant species were distributed across all clades. nsLTP are grouped by type, independently of plant order, suggesting their ancient acquisition prior to the emergence of the RNF clade. Furthermore, we observed several copies of the same nsLTP type in each plant species, suggesting that several duplications took place within each node. This also showed that nsLTPs had an evolutionary history marked by both ancient but also recent duplications in plants and groups of plants. This observation suggests that the *nsLTPs* gain predates the emergence of the RNF clade. This conclusion is consistent with Edstam who argued that nsLTPs would have emerged in the first land plants since no nsLTPs in their dataset were identified in algae at that time^23^. A recent study predicted a novel nsLTP lineage in green alga thus nsLTPs could have emerged in the common ancestor of green plants^24,25^. Our results also showed a high percentage of nsLTPs not corresponding to any of the types proposed by Boutrot et al. but grouped in the same phylogenetic clade with conserved protein motifs. As there is no official classification for these peptides, it would be relevant to complete or establish a new classification of nsLTPs from plants covering the whole plant kingdom and improve the identification of these peptides in proteomes. Studying nsLTPs from plants representing the entire plant kingdom, as they have recently been identified in algae, would also improve analysis of the evolutionary history of nsLTPs^25^.

To deepen our understanding of the evolutionary history of nsLTPs in RNF symbiosis, the expression of genes encoding putative nsLTPs in nodules of 5 plants was retrieved with available transcriptomics data. This permitted the identification of genes encoding putative nsLTPs up-regulated in the functional nodule of plants belonging to the four orders of the RNF clade except for *C. glauca*. The only nsLTP studied in actinorhizal plants targets the nitrogen-fixing vesicle of *F. alni* ACN14a, however, in the symbiosis model between *C. glauca* and *Frankia casuarinae* CcI3, the nsLTPs might not be present in the nodules because *F. casuarinae* Cci3 does not differentiate cells into vesicles in nodule as the oxygen flow is controlled by the host plant^35^. The differential expression data of *CgLTPs* in the functional nodule were not available for all putative nsLTPs, further analysis could improve these data as we have done here for the putative nsLTPs found in the proteome of *A. glutinosa*. The nsLTPs already described in RNF symbiosis in the literature, MtN5, MtnsLTP54 and, AgLTP24 grouped in the same phylogenetic clade and share conserved protein motifs but other putative *nsLTPs* up-regulated in functional nodules had different type and conserved motif and were distributed in all phylogenetic clades. It should also be noted that among the nsLTPs already studied in RNF symbiosis in the literature, AsE246 is so far the only one described as belonging to the type I^19,20^. This raises the question of whether the motifs conserved between MTN5 and AgLTP24 are crucial for their involvement in nodulation, and whether they have the same functions. Furthermore, functional analyses of nsLTPs with different protein motifs belonging to other clades would be required to determine their involvement in nodulation and whether their function is similar or different. Regarding the evolutionary history of nsLTPs in the RNF symbiosis, within the RNF clade, nsLTPs differentially expressed during nodulation belong to different subclades, suggesting that symbiotic functions may have been independently co-opted in different lineages of RNF symbioses. This independent co-option could suggest a convergence of function however, more functional data are required to conclude on this point. It is important to keep in mind that nsLTPs could exhibit the same or diverse functions during the symbiosis. In order to ascertain whether the peptides derived from various nodulating plants, whose encoding genes are up-regulated during symbiosis, possess a single function indicative of functional convergence, or exhibit diverse functions within this association, it is imperative to conduct comprehensive functional studies.

More broadly, concerning the evolutionary history of RNF symbiosis, two hypotheses have been proposed, one with an evolutionary model based on several independent acquisitions^36^ of the ability to form nodulation and another based on a single gain of this trait in a common ancestor followed by multiple losses. Recently, strong arguments have been published supporting the second hypothesis and indicated also that some additional functions have been acquired in a convergent manner such as plant hemoglobin^2–4^. This convergence of function was also described for AMPs involved in RNF symbiosis. NCRs and NCRs-like secreted respectively by legumes belonging to IRLC and Dalbergioids have different structures, but both induce the differentiation of the symbiont into bacteroids with different shapes in the nodule^10,11^. This convergence of function was recently challenged due to their possible origin from within defensins. A recent phylogenetic study between defensins involved in actinorhizal symbioses and NCRs of legumes shows that these peptides would have a common origin^37^. Concerning nsLTPs, their symbiotic functions may have been independently co-opted in different lineages of RNF symbioses to take part of in the symbiosis process in each nodulating plant. That may be a sign of convergent evolution, but it needs to be established first that all *nsLTPs* up-regulated in functional nodule share a common function.

This study focuses only on RNF symbiosis, but it would be worthwhile to determine the involvement of nsLTPs in other mutualistic symbioses such as mycorrhizal symbioses that has not been documented to our knowledge. Only one publication reports the overexpression of a gene encoding nsLTPs in *Oryza sativa* roots during appressoria formation and penetration of the mycorrhizal fungus *Glomus mosseae*. This gene is subsequently down-regulated upon mycorrhization, during the intracellular development of fungal hyphae in the root and is also induced upon treatment with salicylic acid or with the pathogen *Pseudomonas syringae* indicating that this nsLTP is not involved in mycorrhization but probably part of the plant's defense system^38^. In *M. truncatula,* two *nsLTPs* (MtnsLTP104 and MtnsLTP103 corresponding to Medtr4g077180 and Medtr4g076150; respectively) are up-regulated during mycorrhizal symbiosis with the arbuscular mycorrhizal fungi *Rhizophagus irregularis* but no functional studies have been performed. The nsLTPs are described as part of the plant's immune response against many pathogenic organisms such as bacteria, fungi, viruses, nematodes, and insects^39–44^. More broadly this raises the question of how these diverse interactions have shaped the evolution of nsLTPs in plants.

Focusing on RNF interaction, to further investigate the evolution of nsLTPs in nodulating plants, their functions should be explored in nodulating plants of different lineages. This should permit to identify if nsLTPs of a given phylogenetic clade have similar functions or multiple functions during symbiosis. For this purpose, we studied the function of AgLTP24 which is the most expressed nsLTPs gene in the *A. glutinosa* nodule^18^. This peptide inhibits the metabolic activity of *F. alni* ACN14a at 5 µM and inhibits the nitrogen fixation activity at 100 nM, however, the symbiont in the nodule is viable and metabolically active to fix nitrogen to supply the host with nitrogen compound. Thus, in this study, we were interested in the molecular response of the symbiont to subinhibitory concentrations of AgLTP24.

Transcriptomic analysis of *F. alni* ACN14a under N-free conditions supplemented with subinhibitory concentrations of AgLTP24 compared to N-free medium without nsLTP addition indicated that the bacterium copes with stress to ensure its survival by maintaining nitrogen fixation, growth, and respiration and that it was preparing for symbiosis. Several stress-related genes coding for chaperones were up-regulated, as well as genes coding for transporters and transcriptional regulators involved in the management of oxidative stress^45,46^. These genes involved in stress response were also up-regulated at an early step of symbiosis when the bacterium is in indirect contact with the plant^47^. The response of *F. alni* ACN14a to AgLTP24 is comparable to that of the *Sinorhizobium meliloti* symbiont when exposed to NCR247 and NCR335. In both cases, there is an up-regulation of genes encoding heat shock proteins, proteins with potential involvement in transcriptional regulation, and ABC-type membrane transporters^48^. Genes encoding nitrogenase and proteins involved in respiration and the TCA cycle were up-regulated and the gene encoding the nitrite transporter NarK was down-regulated when *Frankia* was in contact with AgLTP24. Under N-free medium in vitro, *F. alni* ACN14a *nif* genes are up-regulated as well as *narK* encoding a nitrite transporter. The *nif* genes are up-regulated and *narK* is down-regulated when the symbiont is in nodule condition compared to an N-free medium^49^. The same expression profile of *nif* and *narK* genes was seen when *Frankia* was in contact with AgLTP24 suggesting that the bacteria had a similar nitrogen management to nodule conditions in planta.

*F. alni* ACN14a in contact with AgLTP24 at subinhibitory concentrations could undergo stress. To overcome this, *Frankia* could establish resistance systems to adapt to the effects of AMPs. Some up-regulated genes encoding ABC transporters that could be a mechanism of resistance to nsLTPs. An ABC transporter, BacA, essential for the survival of the symbiont in the nodule, allows *Sinorhizobium meliloti* to cope with the toxicity of NCR peptides secreted by *M. truncatula*.^50,51^. Two genes encoding peptides possibly involved in succinoglycan synthesis were up-regulated by *Frankia* in contact with AgLTP24. The succinoglycan produced by rhizobia allows them to resist to NCRs^52^. It was described that sub-inhibitory concentration of AMP can act at the membrane or intracellular level, it would be relevant to identify whether this response is induced following the interaction of AgLTP24 with *Frankia* membranes or intracellular target molecules^53^*.*

## Conclusion

Nodulating plants that are co-evolving with their nitrogen-fixing symbionts appear to have independently specialized nsLTPs for this interaction, suggesting a possible convergence of function. To better understand the various functions of these nsLTPs in RNF symbiosis we identified genes encoding putative nsLTPs in plants distributed in the four orders of the RNF clade which opens new perspectives. Concerning actinorhizal symbioses, we confirmed that *AgLTP24* was the most up-regulated gene in the functional nodule of *A. glutinosa* in symbiosis with *F. alni* ACN14a. Thus, the function of this nsLTPs was further investigated with the study of the molecular response of the symbiont to sub-inhibitory concentrations of AgLTP24, which permitted to show a similar response to that found in symbiotic conditions and highlighting possible adaptation mechanisms of *Frankia* to AgLTP24.

## Materials and methods

### nsLTPs identification and characterization

nsLTPs detection was performed using 15 plant proteomes: *Datisca glomerata* (GCA_003255025.1)^2^, *Chamaecrista fasciculata* (GCA_003254925.1)^2^, *Nissolia schottii* (GCA_003254905.1)^2^, *Alnus glutinosa* (GCA_003254965.1)^2^, *Casuarina glauca* (GCA_003255045.1)^2^, *Discaria trinervis* (GCA_003254975.1)^2^, *Dryas drummondii* (GCA_003254865.1)^2^, *Cucumis sativus* PI18396 (PI183967)^54^, *Lupinus albus* (WOCE00000000)^55^, *Medicago truncatula* (PSQE00000000)^56^, *Parasponia andersonii* (GCA_002914805.1)^3^, *Arabidopsis thaliana* (TAIR10, GCA_000001735)^57^, *Juglans regia* (GCF_001411555.2)^58^, *Pyrus communis* (PRJEB5264)^59^ and *Quercus lobata* (GCF_001633185.2).

A wrapper script, nsLTPFinder, was used to identify putative nsLTPs peptides in plant proteomes (https://github.com/jeankeller/nsLtpFinder.git). As input, a directory containing the proteomes was used to be analyzed in FASTA format. First, a HMMSEARCH from the HMMER v3.3 package was performed using the Hidden Markov Model (HMM) profile of Probable lipid transfer (PF14368.6), Hydrophobic seed protein (PF14547.6) and Protease inhibitor/seed storage/LTP family (PF00234.22) (PFAM34 database). Searches were performed using an e-value threshold of 10 for full and domain hits. The nsLTPs were also searched with the regular expression "C.(6,15)C.(6,80)CC.(8,29)C.C.(8,37)C.(4,25)C" in proteomes. Results from HMMSEARCH and the regular expression search were merged and protein sequences were then extracted from proteomes. Signal sequences were searched using SignalP 5.0^60^. The isoelectric point, molecular weight, and grand average hydropathy (GRAVY) were retrieved for peptides and mature peptides, which correspond to the peptides without signal sequence using Expasy ProtParam tool^61^. Proteins identified by the regular expression search and HMMSEARCH with an identified signal peptide and containing 8 cysteines in the mature sequence were extracted as "top candidates" and proteins identified only with the regular expression search with a signal peptide, 8 cysteines in the mature sequence were extracted as low confidence candidates. Conserved motifs were predicted using the MEME^62^ suite on the top and low confidence mature peptides for each plant proteome and on all top and low confidence for all proteomes.

Once nsLTPFinder ran to completion, the 8CMs were manually checked in the mature peptide’s amino acid sequences for top and low-confidence candidates. The identified nsLTPs were grouped according to the classification proposed by Boutrot et al.^20^ and completed with the type XI proposed by Li et al.^30^. The graphical representation of the number of nsLTPs and their classification in each plant was performed using RStudio 2021.09.2. The comparison of the number of putative nsLTP in plants belonging to four orders of the RNF clade and the comparison of the number of nsLTPs present in nodulating and non-nodulating plants belonging to the RNF clade was performed using Shapiro normality test and Mann–Whitney test to analyze the distribution of data using GraphPad Prism 9.5.0.

### Phylogenetic analysis and sequence alignment

Multiple sequence alignments of nsLTPs CDS sequences were performed using Mafft v7 with local pairwise. The alignment was cleaned using TrimAl 1.4.1^63^ to remove positions with more than 50% of gaps. A Maximum-likelihood phylogenetic tree was reconstructed using IQ-TREE2 2.1.4_beta^64^ (SH-aLRT test and ultrafast bootstrap with 10,000 replicates) and the model GTR + F + R9 determined with ModelFinder (10.1038/nmeth.4285) according to the Bayesian Information Criteria. Branch supports were tested using 10,000 replicates of Ultrafast Bootstrap^65^. The tree was visualized with iTOL 6.3.2 platform^66^. The maximum-likelihood phylogenetic tree reconstructed with IQ-TREE2 with bootstrap values in Newick format can be found as Supplementary File S1.

### nsLTPs differential expression during nodulation

To analyze differential expressions of genes encoding nsLTPs in the nodule, previously calculated transcriptomic data from five nodulating plants were recovered from available transcriptomics data. For *M. truncatula*, expression data were obtained after 14 days post-inoculation (dpi) with *Sinorhizobium meliloti* 1021^67^ via the MtSSBPdb platform^32^. Gene annotation correspondence was done using the LeGOO database^33^. The *P. andersonii* expression data were obtained at stage 3 (corresponding to functional nodule) after inoculation with *Mesorhizobium plurifarium* BOR2^3^. Differential expressions data of *D. glomerata* were obtained after 24 dpi with nodule crush^68^. For *C. glauca* the expression data had been obtained on 21 dpi nodules with *Frankia casuarinae* Cci3^69^ using SESAM database^70^.

nsLTPs of *A. glutinosa* (AgLTPs) genes expression in nodule (21 dpi) infected with *F. alni* ACN14a were analyzed using EST (Expressed Sequence Tag) database and microarray analysis, which are publicly available on the Gene Expression Omnibus database (www.ncbi.nlm.nih.gov/geo; accession number GSE24153). Correspondence between EST and *A. glutinosa* gene name^2^ was determined with a Blast search using percentage identity > 90% and EST-gene coverage > 85% parameters. Differential expression of genes encoding AgLTPs was determined using the microarray dataset with a *p*-value threshold of 0.05. Briefly, Student’s *t*-test was applied to compare nodules versus non-inoculated roots and average Fold Changes (FC) were calculated and false discovery rate (FDR) adjusted *p*-value (FC are considered as significative if *p*-value adj < 0.05). To complement and confirm these microarray data, reverse transcription (RT) and quantitative real-time PCR (qRT-PCR) using nodules from 3 plant biological replicates obtained after infection with *F. alni* ACN14a (21 dpi) were performed. The results obtained were compared to uninfected roots as reference. RT was performed using 5 µg of total mRNA using Transcriptor Reverse Transcriptase and oligo (dT)_15_ primer (Roche, Mannheim, Germany). qRT-PCR was run on BioRad QX 100 using iTaq Universal SYBR Green Supermix (Bio-rad) under the following conditions: 95 °C for 5 min; 44 cycles of 95 °C for 20 s, 60 °C for 20 s 72 °C for 15 s. Primer sets were designed using Primer3Plus software and can be found in Supplementary Table S3. Expression values were normalized using the expression level of the *Ag-ubi* gene that encodes ubiquitin^71^.

### Strain and plant growth condition

*Frankia alni* strain ACN14a^72^ was grown at 28 °C with 200 rpm stirring in FBM medium with 5 mM ammonium as described earlier^73^ to the exponential phase. The cells were then harvested, sedimented by centrifugation (5000×*g*, 10 min), and washed twice with corresponding NH_4_^+^-free FBM medium (FBM-). Plant growth, inoculation and nodule harvesting were done as described earlier^13^.

### Molecular response of *F. alni* ACN14a to contact with AgLTP24

AgLTP24 was produced and purified as described earlier^18^. Three independent cultures of *F. alni* ACN14a were made in 240 ml of FBM-liquid medium (N-free condition) supplemented or not with 1 nM of AgLTP24 and grown for 7 days at 28 °C. The pellets were collected by centrifugation at 5100×*g*. Then, mRNAs were extracted and converted into cDNA as described previously^49^. Ribosomal RNAs were depleted using Truseq stranded total RNA (Illumina) and the cDNA were sequenced using Novaseq6000 (Illumina at the MGX, Montpellier, France). Bioinformatic and statistical treatments were made by the MGX (Montpellier GenomiX Platform) platform. The sequences were aligned on the *F. alni* strain ACN14a genome using the BWA 0.7.17-r1188 software^74^. Statistical analyses were made using DESeq2 1.26.0 with R 3.6.1^75^.

Bioassays were conducted by growing *F. alni* strain ACN14a in FBM- and incubating it for 7 days at 28 °C, 3 replicates per condition were performed as described previously^18^. *Frankia’s* nitrogen fixation activity (or ARA), respiration (IRA), and growth (OD_600nm_) were tested as described in previous work^18^. Statistical analyses were computed using RStudio 4.1.2. The normality of the distribution was tested with a Shapiro–Wilk normality test, variances homogeneity was tested with a Fisher’s test. Means comparisons were performed with a Student’s *t*-test. Graphics were made using GraphPad Prism 9.2.0 (GraphPad Software Inc; San Diego, CA, USA).

### Supplementary Information

Supplementary Information.

## Data Availability

The raw reads have been deposited into the European Nucleotide Archive (ENA) (Accession number PRJEB61075).
