# Supplementary material for: Identification and evolution of nsLTPs in the root nodule nitrogen fixation clade and molecular response of Frankia to AgLTP24
Source: Sci Rep. 2023 Sep 25;13:16020. doi: 10.1038/s41598-023-41117-1 (PMC10520049; doi:10.1038/s41598-023-41117-1)
Supplement: Supplementary file 1 — Supplementary Information. [file 41598_2023_41117_MOESM1_ESM.zip › Suplementary datas/Figure S1.pdf]

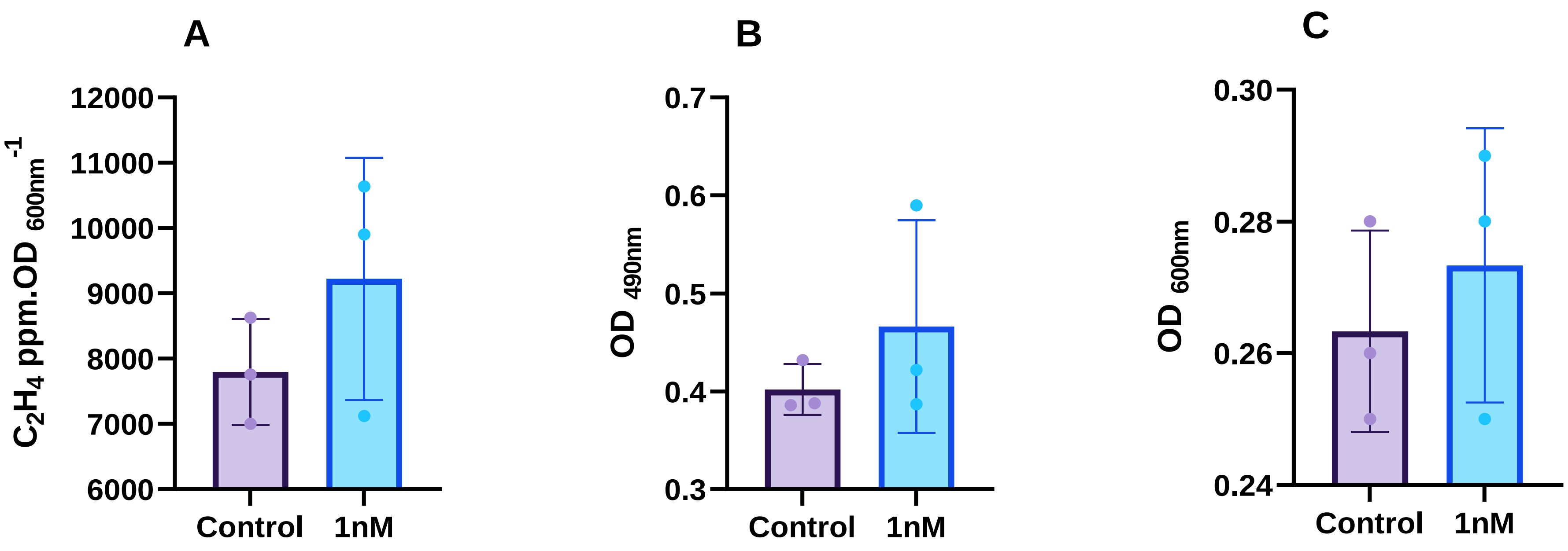

Fig. S1. Kinetics assay of *Frankia alni* ACN14a with a sub-inhibitory concentration of AgLTP24.

A. nitrogen fixation through ARA activity **B.** respiratory activity through IRA and **C.** growth through OD $_{600nm}$ were tested on *Frankia alni* ACN14a cultured in an N-free medium supplemented (1nM) or not (Control) with AgLTP24. The results were obtained after 7 days of growth.

Data are expressed as mean values ± SD. Data normality was tested by the Shapiro-wilk test and variances homogeneity were tested by the Fischer's test. Comparisons of means were assessed by the student t-test. Graphic representation and statistical analysis of results were conducted with GraphPad Prism version 9.2.0 (GraphPad Software Inc; San Diego, CA, USA). *p value <0.05
